# Activation of angiogenin expression in macrophages by lipopolysaccharide via the TLR4/NF-κB pathway in colitis: LPS activates angiogenin expression in macrophages

**DOI:** 10.3724/abbs.2024013

**Published:** 2024-04-03

**Authors:** Zhengrong Yao, Rongpan Bai, Wei Liu, Yaxing Liu, Wei Zhou, Zhengping Xu, Jinghao Sheng

**Affiliations:** 1 Institute of Environmental Medicine and Department of General Surgery Sir Run Run Shaw Hospital Zhejiang University School of Medicine Hangzhou 310058 China; 2 Liangzhu Laboratory Zhejiang University Hangzhou 311121 China; 3 Department of General Surgery Sir Run Run Shaw Hospital .Zhejiang University School of Medicine Hangzhou 310016 China; 4 Cancer Center Zhejiang University Hangzhou 310012 China; 5 Zhejiang Provincial Key Laboratory of Bioelectromagnetics Hangzhou 310058 China

**Keywords:** angiogenin, macrophage, lipopolysaccharide, TLR4, NF-κB

## Abstract

Inflammatory bowel disease (IBD) is a debilitating condition that can lead to life-threatening complications. Macrophages are crucial in IBD management because they secrete various cytokines and regulate tissue repair. Macrophage-derived angiogenin (ANG) has been shown to be essential for limiting colonic inflammation, but its upstream regulatory pathway and role in macrophages remain unclear. Here we show that ANG expression is up-regulated in macrophages during colitis treatment or upon lipopolysaccharides (LPS) treatment. Mechanistically, LPS activates Toll-like receptor 4 (TLR4) to initiate NF-κB translocation from the cytoplasm to the nucleus, where it binds to the ANG promoter and enhances its transcriptional activity, leading to increased ANG expression. Interestingly, our data also reveal that the deletion of ANG in macrophages has no adverse effect on key macrophage functions, such as phagocytosis, chemotaxis, and cell survival. Our findings establish a “LPS-TLR4-NF-κB-ANG” regulatory axis in inflammatory disorders and confirm that ANG controls inflammation in a paracrine manner, highlighting the importance of ANG as a key mediator in the complex network of inflammatory processes.

## Introduction

Inflammatory bowel disease (IBD), which includes Crohn’s disease (CD) and ulcerative colitis (UC), is characterized by chronic and progressive inflammation due to the dysregulation of genetic, environmental, microbial, and immune factors [ 1‒ 4]. This complex interplay contributes to the initiation and development of IBD. Notably, IBD is a leading causative risk factor in the progression of colorectal cancer and has become a major healthcare burden associated with significant global morbidity [ 5, 6]. Thus, a better understanding of the pathophysiological basis and molecular processes involved in IBD could provide us with new perspectives on early diagnosis and therapeutic approaches.

The macrophage-induced innate immune response in the intestinal mucosa serves as the first line of defense against external stimuli [ 7, 8]. Dysregulation of gastrointestinal macrophages, either inefficient or overactivation, contributes to IBD development by modulating the initiation, amplification, and resolution of local inflammation [ 2, 9, 10]. LPS, an outer membrane component of Gram-negative bacteria, functions as a trigger for initiating the inflammatory cascade [ 11, 12]. Binding of LPS to TLR4 activates several key subpathway molecules, such as NF-κB, AP-1 and PI3K, leading to a wide range of cellular responses, including cell differentiation, survival, apoptosis, and inflammatory responses [ 13‒ 15]. In addition, activation of this signaling pathway leads to the production and release of various secreted factors, which play crucial roles in regulating the survival, proliferation, and functions of surrounding cells [ 16‒ 18]. Therefore, understanding the regulatory network of macrophages in both healthy and inflammatory states holds promise for the optimization of IBD therapy.

Angiogenin (ANG) was identified as a novel mediator through which macrophages maintain epithelial barrier integrity and ameliorate intestinal inflammation [19]. By interacting with its receptor, plexin B2 (PLXNB2), on epithelial cells, macrophage-secreted ANG promotes epithelial cell survival and proliferation by regulating tiRNA (tRNA halves) production and rRNA transcription, respectively. Clinically, ANG level is downregulated in colonic tissues from IBD patients, and this change is inversely related to the progression of the disease. Furthermore, the therapeutic potential of recombinant ANG has been demonstrated in a mouse model of colitis. However, the upstream pathway of ANG in macrophages under colitis conditions and its specific role in macrophages have yet to be fully elucidated.

In the present study, we aimed to explore the upstream regulatory mechanisms governing ANG production during the early stages of colitis and following LPS treatment using both *in vivo* and *in vitro* models. Furthermore, we seek to elucidate the consequences of ANG upregulation, focusing on its direct role in macrophage functionality. This study provides a more comprehensive understanding of ANG’s role in intestinal inflammation and its potential as a therapeutic target in the treatment of IBD.

## Materials and Methods

### Mouse breeding and maintenance

*Ang*-knockout (KO) mice were generated by the Guo-fu Hu group at Tufts University/Tufts Medical Center and imported for breeding in our laboratory [20]. For mouse breeding, *Ang*-KO mice and their wild-type (WT) controls were generated from the same heterozygous parents. Mice were maintained and bred under specific pathogen-free conditions with a 12-h light/12-h dark cycle, a 25°C room temperature and 50.0% ± 5.0% humidity at the Laboratory Animal Center of Zhejiang University. Food and water were provided *ad libitum*. Male mice were allocated to experimental groups on the basis of their genotypes and randomized into different treatment groups. All animal studies were performed in compliance with the Guidelines for the Care and Use of Laboratory Animals, and the protocol was approved by the Medical Experimental Animal Care Commission of Zhejiang University (#ZJU20220219).

### Experimental colitis model

Mice were administered dextran sulfate sodium (DSS) in drinking water to induce acute colitis, as previously described [19]. In brief, sex-matched 8-week-old mice were fed with DSS (2.5% w/v; molecular weight: 36 to 50 kDa; MP Biomedicals, Santa Ana, USA) in drinking water for 1 week, followed by regular access to drinking water until the end of the study, after which colonic tissue was collected on the indicated day.

### Isolation of intestinal lamina propria cells

Isolation of intestinal lamina propria cells was performed with an established method reported previously [ 21, 22]. Briefly, after removal of extraintestinal fat tissue and blood vessels, the colons were flushed off of their luminal contents with cold PBS, opened longitudinally, cut into 2-cm pieces, and then incubated with Hank’s balanced salt solution (HBSS) without Ca ^2+^ or Mg ^2+^ containing 5% FBS (Thermo Fisher Scientific, Waltham, USA), 2 mM EDTA (Sigma-Aldrich, St Louis, USA), or 1 mM DTT (Sigma-Aldrich) for 40 min at 37°C with shaking at 250 rpm. After incubation, the tissue was rinsed with ice-cold PBS, and the process was repeated to ensure complete removal of epithelial cells. To release the lamina propria cells, the tissue was further cut into smaller 1-mm pieces and then digested with 1 mg/mL collagenase IV (Sigma-Aldrich) and 0.1 mg/mL DNase I (Roche, Basel, Switzerland) for 40 min at 37°C with shaking at 200 rpm. The digested cell suspension was washed with cold PBS and then passed through 100- and 40-μm cell strainers. After centrifugation at 600 *g* for 5 min, the cells were resuspended in PBS containing 5% FBS for further experiments.

### Cell culture, treatment and transfection

Primary macrophages were isolated and cultured as previously described [23]. Briefly, bone marrow cells were isolated from the femurs and tibias of 8-week-old WT and *Ang*-KO mice. The cells were then cultured in Dulbecco’s modified Eagle’s medium (DMEM; Thermo Fisher Scientific) supplemented with 10% FBS and 20 ng/mL recombinant murine macrophage colony stimulating factor (M-CSF; PeproTech, Waltham, USA) for 5 days to allow differentiation of the cells into BMDMs. Human THP-1 cells are a human monocytic cell line derived from an acute monocytic leukemia patient and were maintained in Roswell Park Memorial Institute medium (RPMI1640; Thermo Fisher Scientific) supplemented with 10% FBS, 1 mM pyruvate (Thermo Fisher Scientific), 2.5 g/L D-glucose (Thermo Fisher Scientific) and 50 pM β-mercaptoethanol (Thermo Fisher Scientific). After 24 h of incubation with 20 μM phorbol 12-myristate 13-acetate (PMA; Sigma-Aldrich), the THP-1 cells were differentiated into macrophages, which were subsequently incubated in RPMI 1640 medium for 24 h. The cells were washed with serum-free RPMI 1640 medium prior to further experiments. All the cells were incubated at 37°C in a humidified 5% CO _2_ atmosphere.

The TLR4 antagonist TAK-242 (MedChemExpress, Monmouth Junction, USA), the NF-κB inhibitor BAY 11-7082 (Sigma-Aldrich), the AP-1 inhibitor T-5224 (Toyama Chemical, Tokyo, Japan), and the PI3K inhibitor Wort (Sigma-Aldrich) were dissolved in dimethylsulfoxide (DMSO; Sigma-Aldrich) prior to use. The cells were pretreated with TAK-242 (1 μM), BAY 11-7082 (20 μM), T-5224 (20 μM), wort (30 μM) or vehicle only (DMSO) for 1 h before being incubated with 100 ng/mL of LPS.

Transfection of siRNA into cells was performed with Lipofectamine RNAiMAX (Thermo Fisher Scientific) according to the manufacturer’s protocol. siRNAs were synthesized by Genepharma (Shanghai, China). The oligonucleotide sequences are listed in Table 1.

**Table:** **Table 1** Sequences of primers and siRNAs used in this study

Name	Sequence (5′→3′)
Mouse gene	
*Ang*	F: CATCCCAACAGGAAGGAAGGA
	R: ACCTGGAGTCATCCTGAGCC
*Actb*	F: GAGCGCAAGTACTCTGTGTG
	R: CGGACTCATCGTACTCCTG
Human gene	
*ANG*	F: AGAAGCGGGTGAGAAACAA
	R: CATAGTGCTGGGTCAGGAAG
*ACTB*	F: AGCGAGCATCCCCCAAAGTT
	R: GGGCACGAAGGCTCATCATT
ChIP assay	
P1	F: GGCTCAGTGAGACACAAGCA
	R: GCCTGCTGTTCTGAAGGTTT
P2	F: ATTCAGAGTGATAGGAAGCT
	R: GGACAGCCCATCGCGAGAC
P3	F: GTAGTCTCTGAAGGGCCGCC
	R: GCTTCTCCGAGGAGGCCCCT
Negative control	F: TACTAGCGGTTTTACGGGCG
	R: TCGAACAGGAGGAGCAGAGAGCGA
siRNA	
siCtrl	UUCUCCGAACGUGUCACGUTT
si *TLR4*-1	GGGCUUAGAACAACUAGAATT
si *TLR4*-2	CCCACAUUGAAACUCAAAUTT

### RNA isolation and qPCR

Total RNA was extracted using Trizol reagent (Thermo Fisher Scientific). The mRNA level of human or mouse ANG was evaluated by reverse transcription (RT) with Moloney murine leukemia virus (M-MLV) reverse transcriptase (Thermo Fisher Scientific), followed by conventional quantitative PCR (qPCR) with SYBR Premix Ex Taq (TaKaRa, Dalian, China). qPCR was performed on a Roche 480 real-time PCR system. *β-Actin* ( *ACTB*) was used as an internal control. The oligonucleotide sequences of primers used are listed in Table 1.

### Protein extraction and immunoblot assay

Protein was isolated from cells using radioimmunoprecipitation assay buffer (RIPA buffer; Beyotime, Shanghai, China) supplemented with protease and phosphatase inhibitors (Thermo Fisher Scientific). The protein concentration was quantified using the BCA assay kit (Beyotime). The extracted protein was separated by SDS-PAGE and transferred onto a polyvinylidene difluoride membrane (Millipore, Billerica, USA). The membrane was blocked with 5% nonfat milk and then incubated with primary antibodies, including anti-ANG antibody (prepared in our own laboratory) and anti-ACTB antibody (#81115-1-RR; Proteintech, Chicago, USA), in TBST (Tris-buffered saline, 0.1% Tween 20) buffer at 4°C overnight. After incubation with HRP-conjugated goat anti-rabbit IgG or HRP-conjugated goat anti-mouse IgG secondary antibody (Thermo Fisher Scientific), the membranes were developed with BeyoECL Star (Beyotime). The chemiluminescent signal intensity in each frame was quantified using ImageJ software (NIH, Bethesda, USA).

### Enzyme-linked immunosorbent (ELISA) assay

Human ANG was measured using an ELISA kit developed in our laboratory [ 24, 25]. Briefly, ELISA plates (Corning, New York, USA) were coated with 1 μg of mouse anti-ANG monoclonal antibody 26-2F (Thermo Fisher Scientific) per well and blocked with 5 mg/mL bovine serum albumin (BSA) in PBS. Cell culture media were added to the wells, and the plates were incubated overnight at 4°C, washed with PBS five times, and incubated with 100 μL of rabbit anti-ANG polyclonal antibody (1 μg/mL, prepared in our own laboratory) for 2 h at room temperature. The wells were washed with PBS four times and incubated with 100 μL of alkaline phosphatase-conjugated goat anti-rabbit IgG antibody (1.25 μg/mL; Thermo Fisher Scientific) for 1 h at room temperature. After the wells were washed with PBS four times, 100 μL of 5 mg/mL p-nitrophenyl phosphate (Thermo Fisher Scientific) in 0.1 M diethanolamine containing 10 mM MgCl _2_ (pH 9.8) was added, and the absorbance was measured at 410 nm. A standard curve of recombinant human ANG was generated at concentrations ranging from 50 to 1000 pg/mL for each plate. The curve was then used to calculate the ANG concentration in each sample.

### Chromatin immunoprecipitation (ChIP)-qPCR

The cells were cross-linked with 1% formaldehyde for 10 min at 37°C, after which the reaction was quenched with 0.125 M glycine. The cell pellets were collected and resuspended in ChIP lysis buffer (50 mM Tris, pH 8.1, 1% SDS, 10 mM EDTA). After sonication to generate DNA fragments 300‒1000 bp in length, the lysates were cleared by centrifugation and diluted 10-fold with ChIP dilution buffer (16.7 mM Tris, pH 8.1, 0.01% SDS, 1.1% Triton X-100, 1.2 mM EDTA, 16.7 mM NaCl). After preclearing with salmon sperm DNA/protein G-agarose at 4°C for 1 h, the samples were incubated with 5 μg of NF-κB IgG (#51-0500; Thermo Fisher Scientific) or control nonimmune IgG (ab171870; Abcam, Cambridge, UK) overnight at 4°C. The immunocomplexes were collected with protein-G-agarose. After incubation with RNase A (Thermo Fisher Scientific) and proteinase K (Thermo Fisher Scientific), DNA was extracted and examined by qPCR with the primers listed in Table 1.

### Luciferase reporter assay

The cells were cotransfected with a pRL-TK plasmid expressing *Renilla* luciferase (as an internal control) and NF-κB binding site or mutant constructs expressing firefly luciferase. Luciferase activities were measured using the Dual-Luciferase Reporter Assay kit (Promega, Madison, USA) 24 h after transfection. The firefly luciferase activity was normalized to the *Renilla* luciferase activity in each sample. The promoter activity of each construct was then normalized to that of the control plasmid pGL3-Basic.

### Phagocytosis of FITC-labelled *E*. *coli*

To assess phagocytosis, FITC-labelled *E*. *coli* was used. Cells were diluted in HBSS at a multiplicity of infection (MOI) of 5:1 or 20:1 and added to BMDMs. After 1 h of incubation, the cells were washed with PBS, and uninternalized bacteria were removed by centrifugation. After three times wash with PBS, the cells were suspended in PBS and analyzed via flow cytometry at 525 nm. A negative control without *E*. *coli* was performed in parallel.

To assess bacterial internalization efficiency, BMDMs were infected with *E*. *coli* at an MOI of 10:1 for 1 h. After incubation, the BMDMs were washed three times with PBS and then incubated for an additional 4 h in 1 mL of DMEM containing 100 μg/mL gentamicin (Thermo Fisher Scientific) to kill any remaining extracellular *E*. *coli*. Subsequently, the BMDMs were washed three times and lysed using 0.1% Triton X-100. The lysates were serially diluted and plated on Tryptic Soy Agar (TSA; BD Diagnostics, Franklin Lakes, USA) plates to enumerate intracellular bacteria.

### Chemotaxis assays

Cell chemotaxis assays were performed using 8-μm pore transwell inserts in a 24-well plate (Corning). The BMDMs were added to the upper chamber, and 600 μL of migration medium supplemented with the chemotactic factors 10 ng/mL M-CSF (Abcam) and 50 ng/mL MCP-1 (Abcam) was added to the lower chamber. The cells were allowed to migrate through the insert membrane for 24 h, 36 h or 48 h at 37°C under a 5% CO _2_ atmosphere. The inserts were then washed with PBS, and nonmigrating cells remaining on the upper surface of the insert were removed with a cotton swab. The migrated cells on the insert were fixed, stained with Diff-Quick (Sigma-Aldrich), and mounted on glass slides. Cell migration was visually measured by counting using a light microscope at 40× magnification. The mean number of cells in 10 randomly chosen fields was calculated for each group. This process was repeated in three separate experiments.

### Cell apoptosis detection

The cells were harvested and subjected to annexin V and PI double staining. After incubation with 5 μL of annexin V-FITC and PI solution (Beyotime) according to the manufacturer’s instructions for 10 min, the cells were immediately analyzed on a flow cytometer (Beckman Coulter, Pasadena, USA).

### Statistical analysis

Data are expressed as the mean±standard deviation (SD). Statistical analysis was performed by Student’s *t* test. Differences were considered to be statistically significant if *P*<0.05. No exclusion criteria were incorporated into the design of the experiments for this study.

## Results

### ANG is upregulated in macrophages during the early stage of colitis

**Figure 1:**
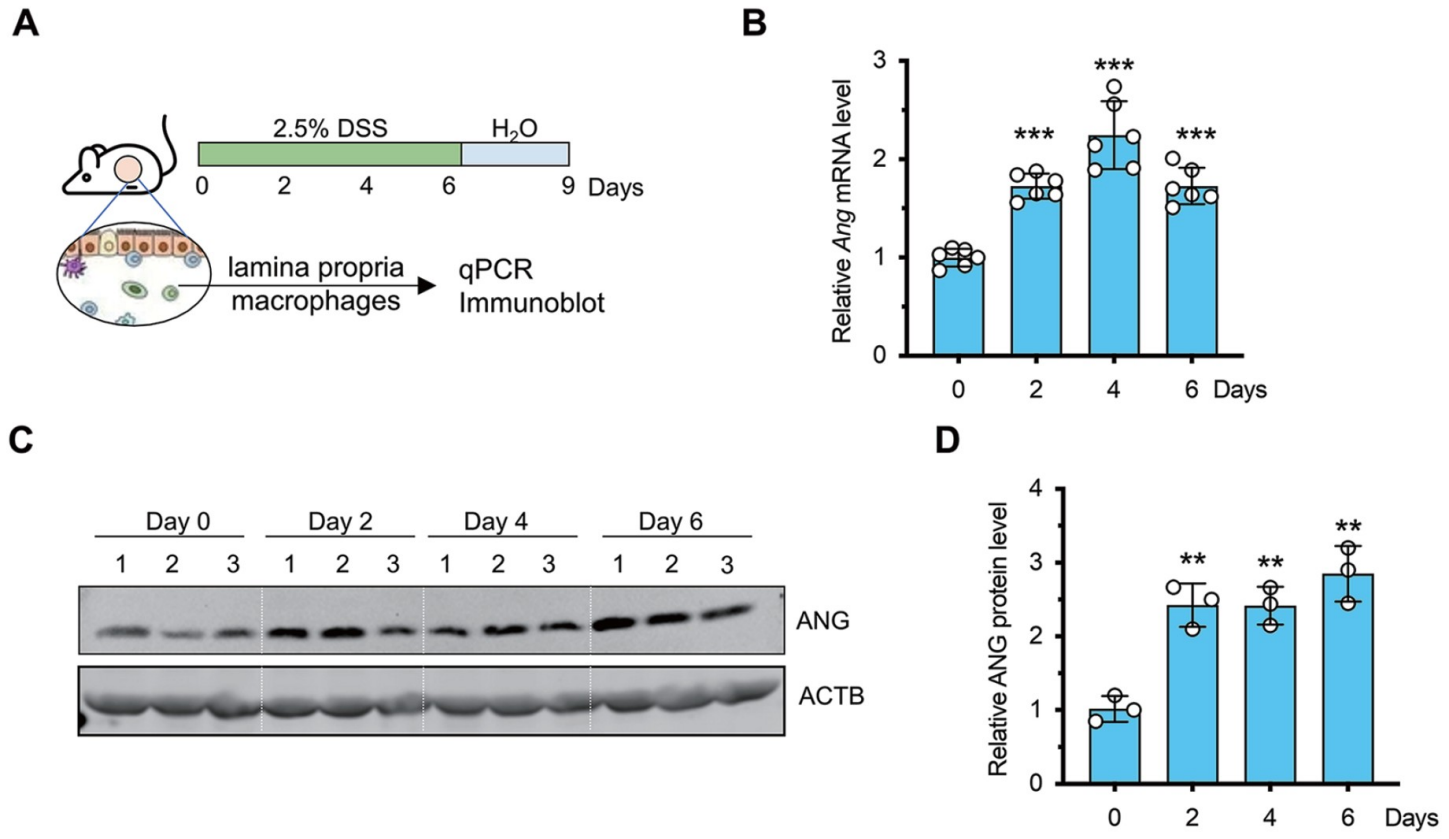
Figure 1 The expression of *Ang* during the early stage of colitis (A) Schematic diagram of the experimental procedure. (B) The mRNA level of Ang on days 0, 2, 4, and 6 of DSS treatment was assessed by qPCR (n=6). (C) Representative immunoblot images. (D) Ang protein levels at 0, 2, 4, and 6 days after DSS treatment, based on grayscale scan calculations (n=3) in panel (C). **P<0.01, ***P<0.001, compared to the day 0 group in (B) and (D).

Although we have identified ANG, which is produced by lamina propria macrophages, as a key factor in controlling colonic inflammation [19], its expression dynamics remain elusive. Therefore, we investigated both the mRNA and protein levels of Ang in lamina propria macrophages isolated from the mouse colon in the context of colitis ( Figure 1A). qPCR analysis revealed gradual upregulation of Ang mRNA expression during experimental colitis, with the highest expression occurring on day 4 after DSS administration ( Figure 1B). In line with these findings, immunoblotting results showed that the ANG protein level increased on day 2 and remained high from day 2 to day 6 after DSS administration ( Figure 1C,D). These findings collectively indicate that ANG expression is upregulated in macrophages during the initial stages of colitis, thereby reinforcing its critical role in managing inflammatory responses.

### LPS-stimulated macrophages produce more ANG

**Figure 2:**
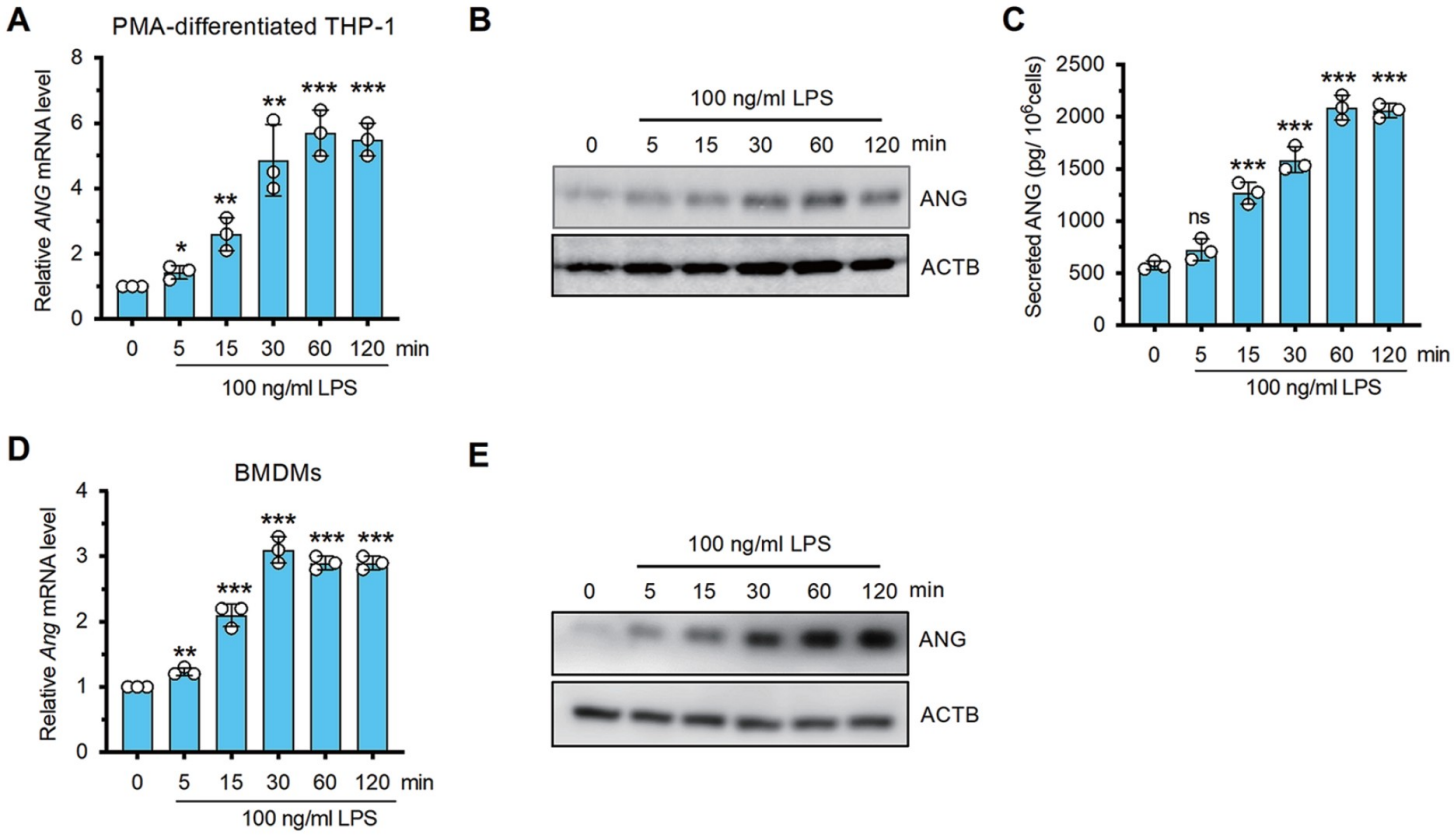
Figure 2 The expressions of ANG in LPS-treated THP-1 cells and BMDMs (A‒C) Time course of PMA-differentiated THP-1 cells treated with 100 ng/mL of LPS for 0, 5, 15, 30, 60 or 120 min, as assessed by qPCR (A), immunoblotting (B) and ELISA (C), respectively. (D,E) Time-dependent Ang expression in murine BMDMs after LPS stimulation, as assessed by qPCR (D) and immunoblotting (E). n=3 for (A), (C) and (D). ns: no significant, *P<0.05, **P<0.01, ***P<0.001, compared to the day 0 group in (A), (C) and (D).

To explore the molecular mechanism underlying ANG expression in both humans and mice, we selected two cell lines, *i.e*., the human cell line PMA-differentiated THP-1 macrophages and murine bone marrow-derived macrophages (BMDMs). Following LPS stimulation, the ANG mRNA and protein levels were detected by qPCR, immunoblotting, and/or ELISA. In PMA-differentiated THP-1 macrophages, we observed dramatic and time-dependent increases in both the mRNA and protein levels of ANG after 120 min of LPS administration. The mRNA and protein levels increased gradually, reaching a maximum at 60 min ( Figure 2A‒C). In BMDMs, the change in Ang expression pattern induced by LPS administration was similar to the change in Ang expression in PMA-differentiated THP-1 macrophages ( Figure 2D,E). These data suggest that ANG increases in response to LPS stimulation in macrophages.

### The TLR4/NF-κB pathway is involved in LPS-induced ANG expression

**Figure 3:**
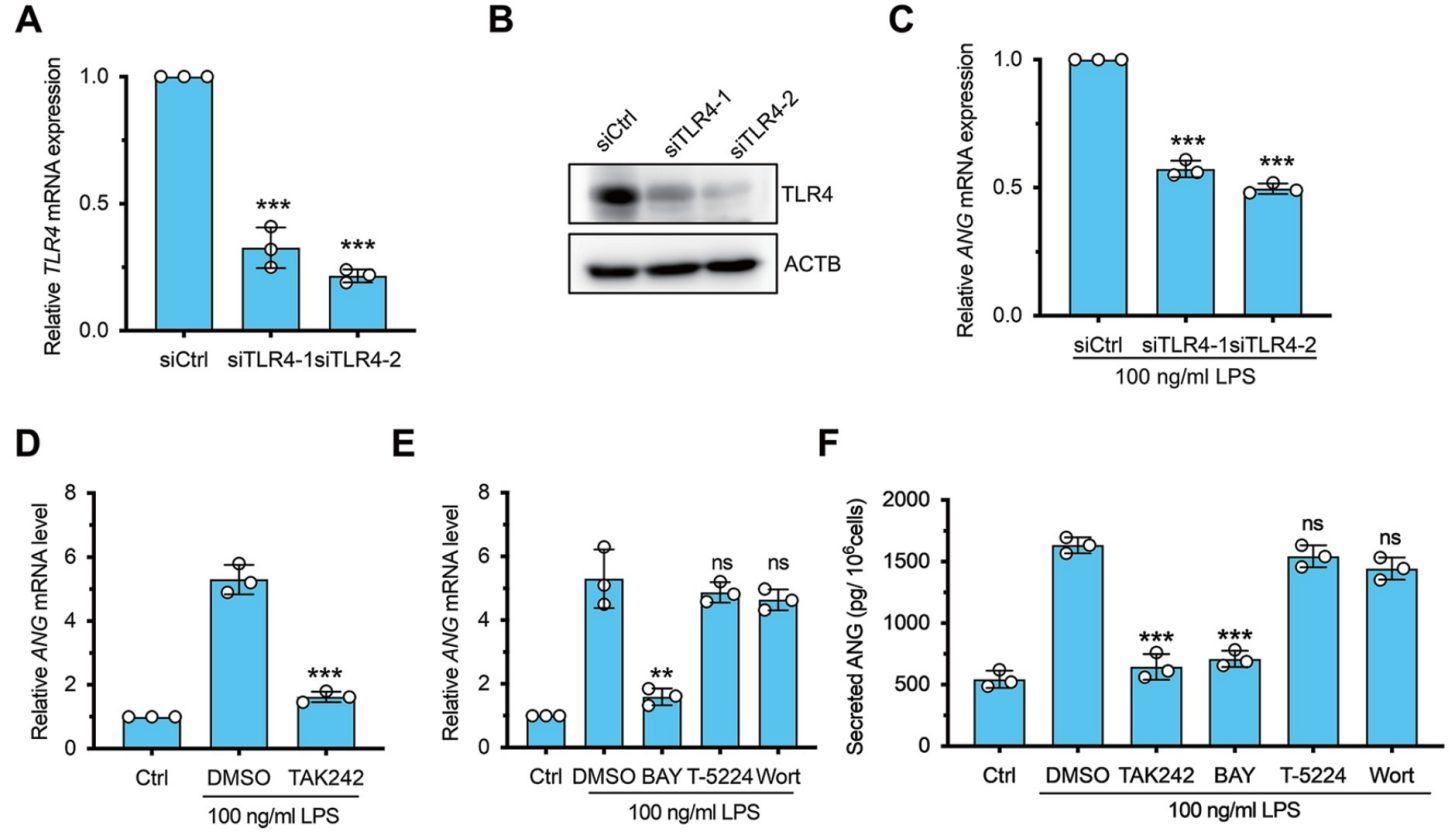
Figure 3 The role of the TLR4/NF-κB pathway in LPS-induced ANG expression (A) qPCR analysis of TLR4 mRNA expression in PMA-differentiated THP-1 cells transfected with TLR4 siRNAs (siTLR4-1 and siTLR4-2) or negative control siRNA (siCtrl) for 48 h (n=3). (B) Immunoblot analysis of the TLR4 protein in PMA-differentiated THP-1 cells transfected with TLR4 siRNAs or the control siRNA. (C) ANG mRNA expression in TLR4-silenced PMA-differentiated THP-1 cells treated with LPS. (D) ANG mRNA expression in PMA-differentiated THP-1 cells not treated with LPS (ctrl) or pretreated with DMSO or TAK242 (a TLR4 inhibitor) followed by LPS treatment (n=3). (E) ANG mRNA expression in PMA-differentiated THP-1 cells not treated with LPS (ctrl), pretreated with DMSO, BAY 11-7082 (an NF-κB inhibitor), T-5224 (an AP-1 inhibitor), or wortmannin (a PI3K inhibitor), followed by LPS treatment (n=3). (F) ELISA analysis of secreted ANG protein in the culture supernatant of PMA-differentiated THP-1 cells treated with pathway-specific inhibitors and LPS (n=3). ns: no significant, **P<0.01, ***P<0.001, compared to the siCtrl group in (A) and (C) and the DMSO group in (D), (E) and (F).

TLR4, the receptor of LPS, is a type of transmembrane protein found on various immune cells, including macrophages [26]. The binding of LPS to TLR4 triggers an intracellular signaling cascade that initiates an innate immune response [27]. To investigate the role of TLR4 in ANG induction by LPS, we silenced endogenous TLR4 using siRNAs. qPCR and immunoblotting confirmed that TLR4 was efficiently knocked down after 48 h of siRNA transfection in PMA-differentiated THP-1 cells ( Figure 3A,B). After administration with LPS, the *ANG* mRNA level was significantly lower in the *TLR4* siRNA-treated cells than in the siRNA negative control cells ( Figure 3C). To further confirm the involvement of *TLR4* in LPS-induced *ANG* expression, we treated cells with TAK242, an inhibitor of TLR4, and found that TAK242 significantly decreased *ANG* expression ( Figure 3D). Several key subpathways are involved in classical LPS/TLR4 signal transduction, such as the NF-κB pathway, MAPK/AP-1 pathway and PI3K/Akt pathway [28]. However, the specific pathway responsible for ANG regulation is still not yet understood. Thus, the NF-κB inhibitor BAY 11-7082 (BAY), the AP-1 inhibitor T-5224 and the PI3K inhibitor wortmannin (Wort) were used to determine the exact pathway that regulates *ANG* mRNA transcription in response to LPS stimulation. The data showed that only pretreatment with the NF-κB inhibitor BAY markedly inhibited *ANG* mRNA accumulation in PMA-differentiated THP-1 cells following LPS administration ( Figure 3E), indicating that NF-κB is involved in LPS-induced *ANG* expression. The ELISA results were in agreement with the qPCR results, further supporting the notion that the TLR4/NF-κB pathway plays a significant role in the increase in ANG induced by LPS ( Figure 3F).

**Figure 4:**
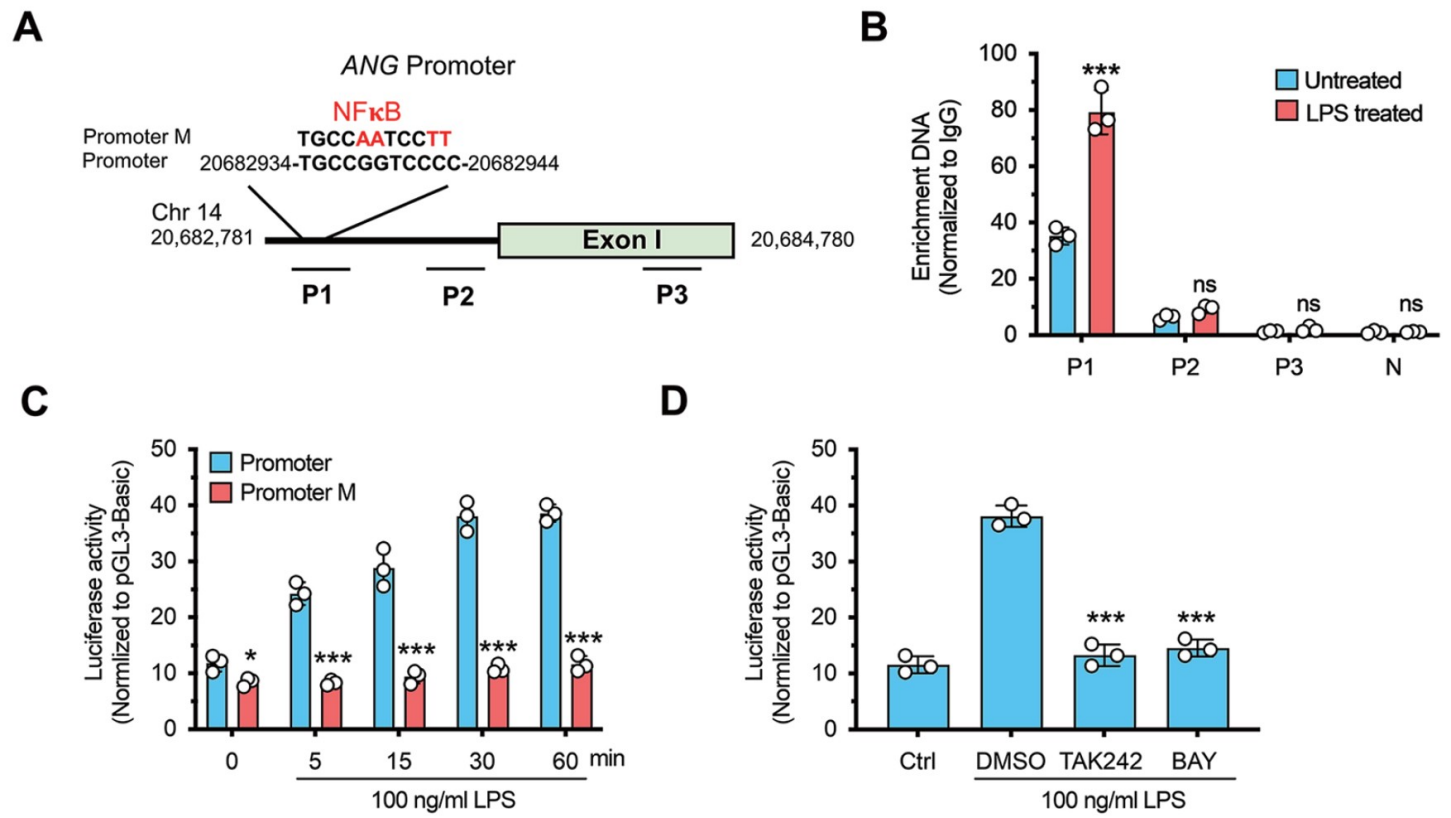
Figure 4 NF-κB binds to the *ANG* promoter and regulates its transcription in response to LPS stimulation (A) Schematic representation of the putative NF-κB binding motif identified in the ANG gene promoter region (Chr 14: 20,682,781‒20,684,780) by bioinformatics sequence analysis. (B) ChIP-qPCR assay showing enrichment of NF-κB at the predicted NF-κB binding site (P1) in the ANG promoter region in PMA-differentiated THP-1 cells (n=3). (C) Luciferase reporter assay of ANG promoter activity (n=3). (D) Effect of TAK242 and BAY pretreatment on ANG promoter-driven luciferase activity (n=3). ns: no significant, *P<0.05, ***P<0.001, compared to the untreated group in (B), the promoter group in (C) and the DMSO group in (D).

In our previous study, we identified putative NF-κB binding motifs within the *ANG* gene promoter region (Chr 14: 20,682,781‒20,684,780) through bioinformatics sequence analysis [24] ( Figure 4A). To explore whether this motif is an NF-κB binding site, we used ChIP-qPCR assay to validate the binding in PMA-differentiated THP-1 cells after LPS treatment. Chromatin from LPS-treated or untreated cells was sonicated and immunoprecipitated with an anti-P65 (subunit of NF-κB) antibody; only one fragment containing the putative NF-κB binding element (P1) was enriched, and its abundance increased in response to LPS administration ( Figure 4B). We then assessed the role of NF-κB in regulating ANG promoter activity using a dual luciferase reporter system. The results showed that the *ANG* promoter induced a substantial increase in basal luciferase activity compared to that of the promoter-free luciferase construct (pGL3-Basic) ( Figure 4C). LPS enhanced luciferase activity in a time-dependent manner. However, this upregulation was not detected in the NF-κB binding site-mutated promoter (promotor M) group ( Figure 4C). Furthermore, pretreatment with the TLR4 inhibitor TAK242 and the NF-κB inhibitor BAY markedly inhibited LPS-induced luciferase activity ( Figure 4D). Collectively, these data further support that upon LPS stimulation, TLR4 is activated to induce nuclear translocation of NF-κB, thus resulting in binding of NF-κB to a specific site within the *ANG* promoter and enhancing *ANG* transcription.

### ANG does not affect macrophage phagocytosis or chemotaxis or cell survival

**Figure 5:**
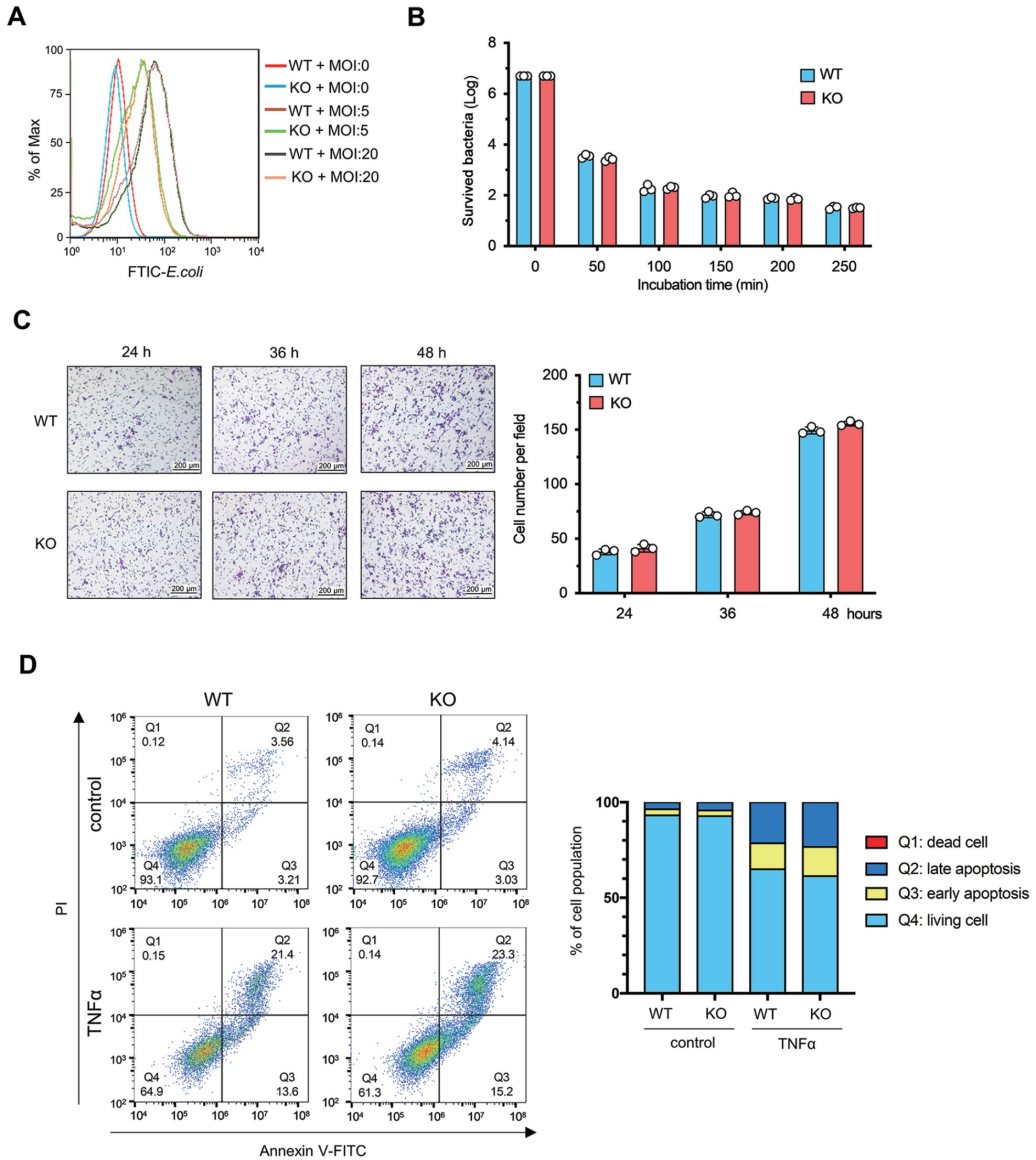
Figure 5 *Ang* deletion in macrophages does not affect phagocytosis, chemotaxis, or cell survival *in vitro* (A) Flow cytometry analysis of FITC-labelled E. coli uptake by KO and WT BMDMs after 1 h of exposure to FITC-conjugated E. coli at an MOI of 5 or 20. (B) Plate count assessment of bacteria killed by KO and WT BMDMs after 1 h of exposure to E. coli at an MOI of 5 (n=3). (C) Chemotaxis in KO and WT BMDMs analyzed by a transwell assay (n=3). The left panel shows a representative image, and the right panel shows the quantification. Scale bar: 200 μm. (D) Flow cytometry analysis of apoptosis in KO and WT BMDMs after TNFα treatment using annexin V and propidium iodide (PI) staining (n=3).

Our previous data showed that ANG does not affect cytokine production or release by macrophages [19]. Here, we examined whether ANG could impact other macrophage functions, such as phagocytosis, chemotaxis and cell survival. First, we assessed macrophage phagocytosis. *Ang*-knockout (KO) or wild-type (WT) BMDMs were exposed to FITC-labelled E. coli at a multiplicity of infection (MOI) of 5 or 20 for 1 h, and cellular uptake was assayed by flow cytometry, while bacterial killing was assessed via plate counting. Both KO and WT macrophages were capable of phagocytosing and killing *E. coli*, but the difference was not significant ( Figure 5A,B). Second, we explored the influence of ANG on macrophage chemotaxis. KO and WT BMDMs were harvested and seeded in the upper chamber of a transwell plate with a lower chamber containing M-CSF and MCP1. The cells were allowed to migrate for 24, 36 or 48 h, and cell migration was quantified by measuring the optical density of the stained cells. Both the KO and WT BMDMs migrated into the transwells, but the difference between the two groups was not statistically significant ( Figure 5C). Third, we evaluated the degree of apoptosis in KO and WT BMDMs after the administration of TNFα by flow cytometry with annexin V and propidium iodide (PI) staining. Compared to the control, TNFα-treated cells showed increased apoptosis; however, the apoptotic cells remained unaltered in KO BMDMs compared to WT ones ( Figure 5D). Taken together, these findings suggest that Ang deletion in macrophages does not affect phagocytosis, chemotaxis or cell survival *in vitro*.

## Discussion

**Figure 6:**
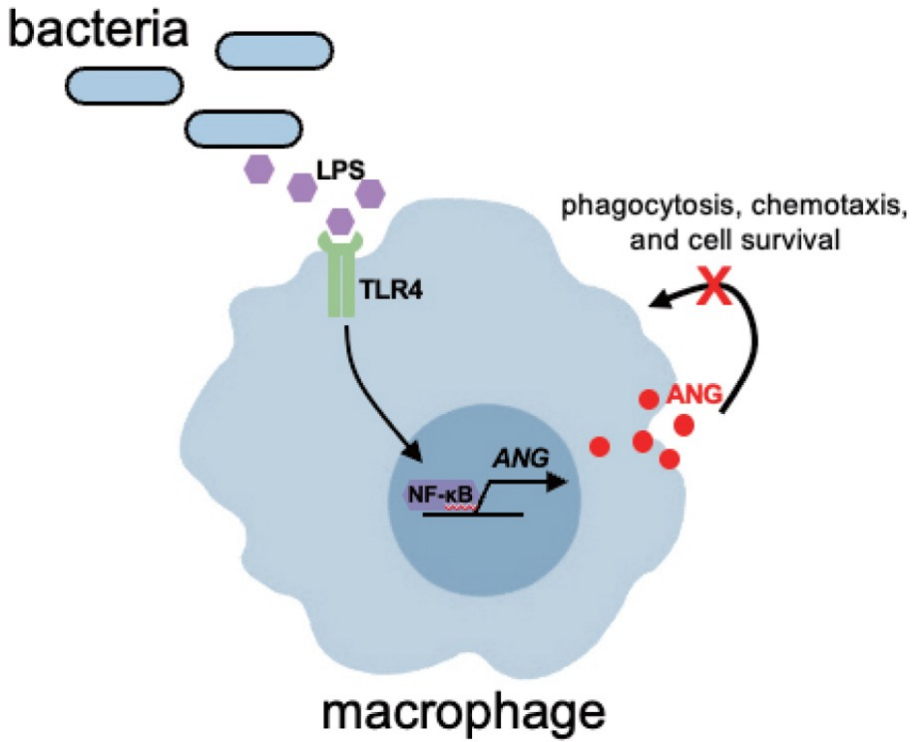
Figure 6 Schematic representation of ANG expression regulation and its role in macrophages LPS activates the TLR4 signaling pathway, leading to the nuclear translocation of NF-κB and binding to the ANG promoter, promoting ANG gene transcription in macrophages. However, ANG does not significantly impact macrophage phagocytosis, chemotaxis, or cell survival.

In the present study, we investigated the relationship between ANG expression and the TLR4/NF-κB signaling pathway in macrophages using LPS as a stimulus ( Figure 6). Our data indicate that upon LPS induction, TLR4 in macrophages is activated, followed by NF-κB nuclear translocation and binding to a specific site in the *ANG* promoter, thus activating *Ang* transcription. Moreover, we also found that ANG does not affect macrophage phagocytosis, chemotaxis, or survival. Overall, this study establishes a mechanism governing ANG expression in macrophages in inflammatory disorders and illustrates that ANG has little influence on the functions of macrophages themselves.

Although our understanding of the pathogenesis of IBD has significantly advanced in recent years, the crosstalk between immune cells and intestinal epithelial cells and the underlying molecular mechanisms involved have not been fully elucidated [ 29, 30]. A previous study revealed that ANG is a crosstalk mediator that maintains epithelial barrier integrity and that its expression is altered during IBD development [19]. However, the precise regulatory events governing ANG expression during the inflammatory response have not been fully elucidated. In this study, we delineated the upstream pathway regulating ANG expression in macrophages, thereby providing a novel approach for manipulating ANG level, which might be beneficial for strategy development in IBD therapy.

Our findings indicate that activation of the NF-κB pathway is the main regulatory mechanism involved in ANG expression. The NF-κB pathway is well known for its role in orchestrating immune responses and controlling the production of inflammatory cytokines and chemokines, such as TNF-α, IL-1β, and IL-6 [31]. The preferential utilization of the NF-κB pathway by ANG aligns with its established function in maintaining the integrity of the epithelial barrier, which is critical in the early stages of colitis [ 19, 32]. Moreover, our results suggest that the MAPK and PI3K/Akt pathways, which are typically associated with bolstering the production of proinflammatory cytokines [ 12, 33] and the direct functions of macrophages [ 34‒ 36], respectively, do not contribute to ANG expression regulation. However, these pathways are still integral parts of the intricate network of immune signaling pathways and play crucial roles in diverse aspects of the immune response. It is possible that under different conditions or contexts, these pathways may trigger ANG expression. Therefore, further studies are needed to explore these potential interactions and their implications.

In this study, we noted that LPS induced ANG expression in both mouse BMDMs and THP-1-derived macrophages. The mechanistic investigations primarily focused on THP-1 cells due to their greater suitability for genetic manipulation. However, we recognize the critical differences in macrophage biology across species, as well as between monocyte-derived macrophages and those generated from pro-monocyte cell lines, in terms of marker expression, signaling pathways, and the production of bioactive factors [ 37, 38]. Therefore, these findings provide a foundation for future studies but necessitate cautious interpretation when considering their application to human biology.

Our findings further support the idea that macrophage-derived ANG primarily acts in a paracrine manner to influence the surrounding environment [19], particularly intestinal epithelial cells, rather than directly modulating macrophages themselves. This finding is also in line with previous findings that PLXNB2, the receptor for ANG, does not play a significant role in regulating macrophage functions such as cytokine production, phagocytosis, migration toward chemoattractants, and the extracellular matrix [39]. The lack of significant effects of ANG on macrophages, despite the expression of its receptor PLXNB2, raises intriguing questions about whether specific inhibition pathways are involved in ANG signaling within this cell type. It would be interesting to investigate whether there are additional regulatory mechanisms or antagonistic factors downstream of the PLXNB2 pathway in macrophages that counteract ANG function.

In summary, our study demonstrated that LPS induces ANG expression in macrophages through the TLR4/NF-κB pathway and that LPS has no autocrine effects on macrophages. Our findings contribute to a better understanding of immune regulation and may pave the way for developing novel therapeutic approaches targeting ANG and its associated pathways to cure inflammatory disorders.
